# Subsequent treatment strategies following rituximab-resistance in AQP4-IgG+ neuromyelitis optica spectrum disorder: a case series

**DOI:** 10.3389/fimmu.2026.1762535

**Published:** 2026-04-16

**Authors:** Meiqun Deng, Lei Chen, Wei Chen, Keyi Zeng, Yuxin Yao, Hanfei Chen, Aiyu Lin

**Affiliations:** 1 Department of Neurology, The First Affiliated Hospital, Fujian Medical University, Fuzhou, China; 2 Department of Neurology, The First Clinical College of Fujian Medical University, Fuzhou, Fujian, China; 3 Fujian Institute of Neurology, the First Affiliated Hospital, Fujian Medical University, Fuzhou, China; 4 Department of Neurology, National Regional Medical Center, Binhai Campus of the First Affiliated Hospital, Fujian Medical University, Fuzhou, China

**Keywords:** case report, neuromyelitis optica spectrum disorder, relapse, rituximab, treatment

## Abstract

**Objective:**

This case series describes alternative treatments for adults with aquaporin 4 immunoglobulin G-seropositive (AQP4-IgG+) neuromyelitis optica spectrum disorder (NMOSD) who exhibited disease activity despite having sustained B cell depletion for more than six months post-rituximab (RTX), a condition we defined as RTX resistance.

**Methods:**

We conducted a single-center, retrospective case series of 10 AQP4-IgG-positive NMOSD patients who met our definition of RTX resistance and subsequently switched therapies. Among the 10 patients, one was switched to the anti-CD20 monoclonal antibody ofatumumab, two to the anti-CD19 monoclonal antibody inebilizumab, two to the IL-6 receptor antagonist satralizumab, and one to the C5 complement inhibitor eculizumab, while 4 transitioned to de-escalations (mycophenolate mofetil, intravenous immunoglobulin, or steroids).

**Results:**

Over a median follow-up of 28.4 months after treatment switch, no relapses were observed among patients who received inebilizumab, satralizumab, or eculizumab. In contrast, the patient who switched to ofatumumab experienced relapse, and three of the four patients (75%) on de-escalations had at least one relapse. No significant adverse events occurred in patients treated with ofatumumab, inebilizumab, satralizumab, or eculizumab, while two serious adverse events were reported among those on de-escalation regimens.

**Conclusion:**

In this descriptive case series of 10 patients with RTX-resistant NMOSD, those who switched to inebilizumab, satralizumab, or eculizumab appeared to have fewer relapses and a favorable safety profile compared to those receiving de-escalation strategies or ofatumumab. These real-world observations provide hypothesis-generating data that may inform clinical decision-making and warrant validation in larger, prospective cohorts.

## 1 Introduction

Neuromyelitis optica spectrum disorder (NMOSD) is an autoimmune disease of the CNS characterized by the production of disease-specific autoantibodies against aquaporin-4 (AQP4) water channels (1). Given the fundamental role of B cells in its pathogenesis, the anti-CD20 monoclonal antibody rituximab (RTX) has emerged as a first-line therapy based on evidence from multiple clinical studies (2). Through B cell depletion, RTX reduces relapses and slows relapse-associated disability accrual (3), while its sustained activity—persisting for months after serum clearance—provides a unique pharmacokinetic profile conducive to long-term disease control (4). However, a subset of patients exhibits treatment failure to RTX (5–7). Notably, a distinct subgroup of patients experienced relapse despite sustained B cell depletion (proportion of CD19^+^ B cells within peripheral blood mononuclear cells at relapse < 1% and CD27^+^ memory B cells < 0.05%) following more than six months of RTX therapy. Based on this observation, we characterize these cases as “RTX-resistant”, thereby proposing a novel definition predicated on breakthrough disease activity under complete B cell depletion, in contrast to the conventional RTX-refractory relapse focused on early relapse within 6 months. Within our institutional cohort of 118 AQP4-IgG-positive patients treated with RTX, 10 (8.5%) met this definition.

While recent consensus statements have proposed the use of newer monoclonal antibodies such as Eculizumab, Inebilizumab, and Satralizumab, definitive evidence remains limited (8–10). However, current guidance relies heavily on isolated reports and small series, therefore the management of RTX-resistant NMOSD remains a significant clinical dilemma, underscoring the need for larger studies to comprehensively evaluate clinical outcomes.

Therefore, this study presents a single-center, retrospective case series of 10 patients with NMOSD who met the criteria for RTX resistance and subsequently received alternative therapies. We describe the clinical characteristics, treatment strategies, and outcomes of these patients, providing real-world, hypothesis-generating observations that may inform future research and clinical decision-making in this challenging setting.

## 2 Methods

### 2.1 Patient identification and selection

**Figure 1:**
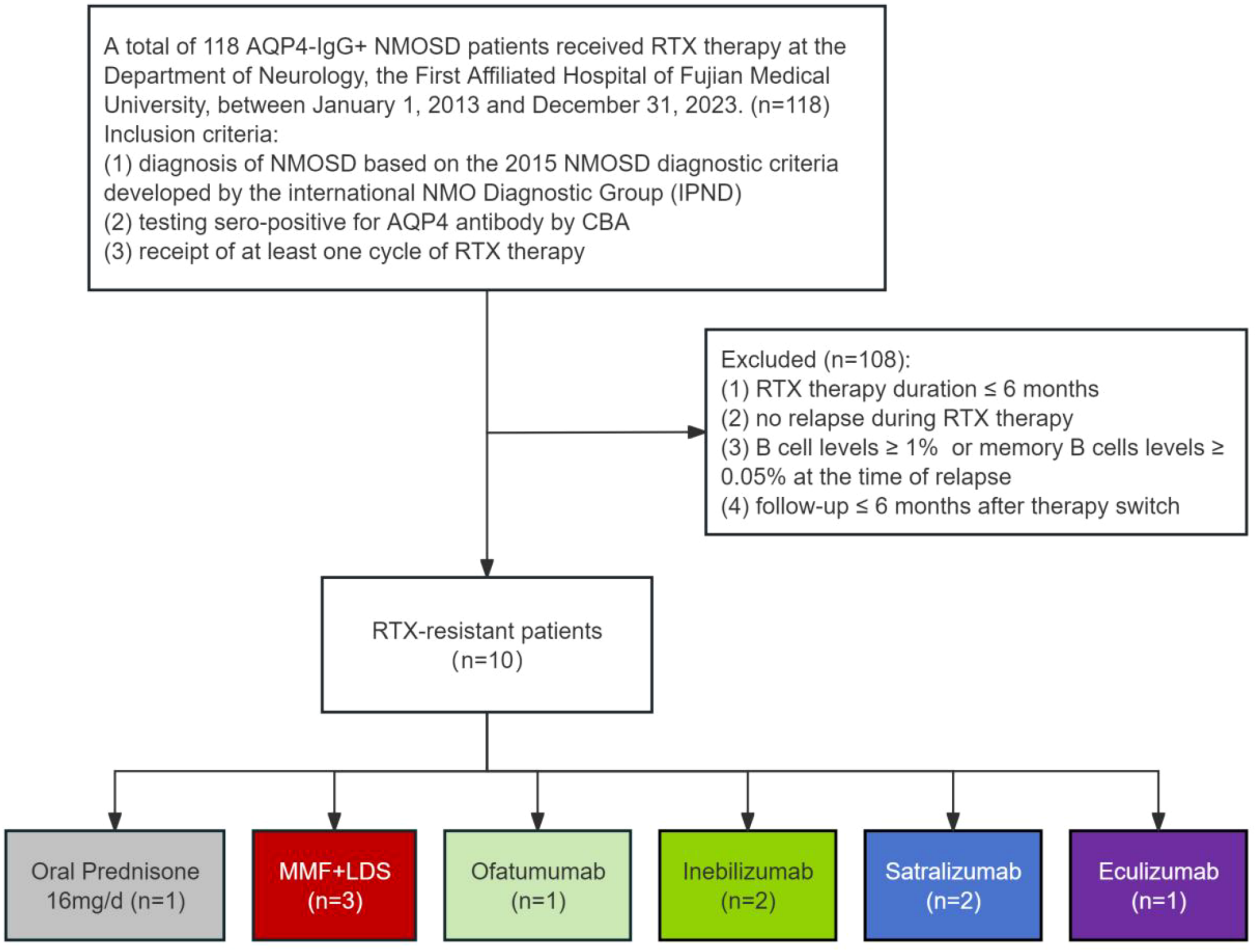
Patient selection and treatment allocation. NMOSD, neuromyelitis optica spectrum disorder; RTX, rituximab; B cell level, Proportion of B cells within peripheral blood mononuclear cells; Memory B cell level, Proportion of memory B cells within peripheral blood mononuclear cells; LDS, Low-dose Steroids (4 mg per day of oral prednisone); MMF, Mycophenolate Mofetil; IVIg, Intravenous Immunoglobulin.

We conducted a retrospective case series at the First Affiliated Hospital of Fujian Medical University. Electronic medical records were interrogated to identify all patients with a diagnosis of NMOSD who had received at least one cycle of RTX between January 1, 2013 and December 31, 2023. The following were the inclusion criteria: (1) diagnosis of NMOSD based on the 2015 NMOSD diagnostic criteria developed by the International Panel for NMO Diagnosis (IPND); (2) testing sero-positive for AQP4 antibody (confirmed by cell-based assay [CBA]); (3) receipt of at least one cycle of RTX therapy. A total of 118 patients were identified. Patients were classified as RTX-resistant if they experienced relapse despite sustained B cell depletion (proportion of CD19^+^ B cells within peripheral blood mononuclear cells at relapse < 1% and CD27^+^ memory B cells < 0.05%) after more than six months of RTX therapy. Relapses were defined as new CNS symptoms and signs lasting longer than 24 h, with or without an associated new lesion on gadolinium-enhancing MRI. Exclusion criteria were: (1) testing sero-negative for AQP4 antibody (CBA); (2) myelin oligodendrocyte glycoprotein antibody-associated disease (MOGAD) or multiple sclerosis (MS); (3) RTX therapy duration ≤ 6 months; (4) no relapse during RTX therapy; (5) B cell level ≥ 1% or memory B cell level ≥ 0.05% at the time of relapse; (6) follow-up ≤ 6 months after therapy switch. A total of 10 (8.5%) patients who met the predefined criteria for RTX resistance were included in the final analysis. The patient selection process is summarized in **Figure 1**.

### 2.2 Data collection

For each patient, we retrospectively collected the following data from their medical records: (1) demographics and clinical characteristics: including sex, core clinical characteristics, AQP4-IgG serostatus, FCGR3A-V158F genotype, serological status of other relevant accompanying autoantibodies and comorbidities; (2) Clinical timeline: including age at key disease milestones, RTX regimen, duration of RTX therapy, potential precipitating factors for relapse, B cell (CD45^+^CD19^+^) levels at relapse, memory B cells (CD19^+^CD27^+^CD38^dim^) levels at relapse, serum IL-6 levels, alternative therapies after RTX and follow-up duration after switch, time to B cell re-appearance (≥ 1%) and adverse events; (3) EDSS scores and annualized relapse rate (ARR) before and after therapy switch.

### 2.3 Standard protocol approvals, registrations, and patient consents

This retrospective study was reviewed and approved by the Ethics Committee of the First Affiliated Hospital of Fujian Medical University (Approval Number (2025):179). Due to the retrospective nature of the study, the requirement for written informed consent was waived. All patient data were anonymized and handled in accordance with institutional and international ethical standards to ensure confidentiality and data protection. The patients included in this analysis were derived from a registered clinical cohort (NCT04386018).

## 3 Case reports

We describe a case series of 10 AQP4-IgG-seropositive NMOSD patients who transitioned to alternative therapies after experiencing relapse while maintaining profound B cell depletion (proportion of CD19^+^ B cells within peripheral blood mononuclear cells at relapse < 1% and CD27^+^ memory B cells < 0.05%) on RTX. This case series (80% female) included patients with the following core clinical characteristics: optic neuritis in 4 patients, transverse myelitis in 1, and combined optic neuritis and transverse myelitis in 5. Given previous reports suggesting a relationship between FCGR3A genetic polymorphisms and RTX response in NMOSD (11), we performed FCGR3A genotyping in 8 of the 10 RTX-resistant patients. The distribution of FCGR3A-V158F genotypes was as follows: three patients were homozygous for the F allele (F/F), three were homozygous for the V allele (V/V), and two were heterozygous (V/F). In this small series, no clear association was observed between specific FCGR3A genotypes and the timing or pattern of RTX failure; however, this finding should be interpreted with caution given the limited sample size. Accompanying autoantibodies were detected in 6 patients. Comprehensive characteristics data are presented in **Table 1**.

**Table 1:** Demographics and clinical characteristics of the 10 RTX-resistant NMOSD patients.

Patient	Gender	Core clinical characteristics	AQP4-IgG serostatus	FCGR3A	Accompanying antibodies	Comorbidities
1	F	ON	Positive (1:100)	VF	ANA+, ACA+, TPOAb+	Hyperthyroidism, Vitiligo
2	F	ON+TM	Positive (1:32)	VF	None	Hypercholesterolemia
3	F	ON	Positive (1:100)	FF	AHA(+-)	None
4	M	ON+TM	Positive	FF	None	Hypercholesterolemia, Nephrolithiasis
5	F	TM	Positive (1:100)	Unknown	TRAb+, TPOAb+	Hypercholesterolemia
6	F	ON+TM	Positive (1:32)	VV	AHA+, TGAb+, TPOAb+, Anti-Ro-52+	Multinodular goiter
7	F	ON+TM	Positive (1:320)	Unknown	None	None
8	F	ON+TM	Positive (1:100)	VV	TGAb+, TPOAb+, ACA+	Hypercholesterolemia, Moyamoya disease
9	F	ON	Positive (1:32)	VV	ANA+, AHA+	Hypercholesterolemia
10	M	ON+TM	Positive	FF	None	Hypercholesterolemia, Nephrolithiasis

RTX, rituximab; NMOSD, neuromyelitis optica spectrum disorder; F, Female; M, Male; ON, optic neuritis; TM, transverse myelitis; ANA, antinuclear antibody; ACA, anticardiolipin antibody; AHA, antihistone antibody; TPOAb, thyroid peroxidase antibody; TRAb, thyrotropin receptor antibody; TGAb, thyroglobulin antibody; Anti-Ro-52, anti-Ro-52 antibody.

All patients received RTX according to one of three protocols: (1) 100 mg on day 1 followed by 500 mg on day 2 (100 mg + 500 mg); (2) 100 mg on three consecutive days (100 mg × 3); or (3) a single dose of 500 mg. Re-treatment was guided by B cell monitoring: RTX was re-administered if the proportion of CD19^+^ B cells among peripheral blood mononuclear cells exceeded 1% or if CD27^+^ memory B cells exceeded 0.05%; otherwise, re-treatment was scheduled at fixed six-month intervals. The median (range) of RTX therapy duration was 32 (6-108) months (**Table 2**). At relapse during RTX therapy, B cell levels (CD45^+^CD19^+^) remained below 1% in all patients. Memory B cell levels (CD19^+^CD27^+^CD38^dim^) were below 0.05% in 7 patients, whereas levels in the remaining 3 were not assessed. Among the 10 patients, MRI data were available for seven (**Supplementary Table 1**; **Supplementary Figure 1**). Only one patient (Patient 9) exhibited asymptomatic MRI lesions, presenting new abnormal enhancement at the C2–3 level without corresponding clinical symptoms at the time of imaging; however, the patient developed neck pain a month later, consistent with the report by Marrodan et al. that asymptomatic MRI lesions during RTX therapy are a strong predictor of therapeutic failure in AQP4-IgG-positive NMOSD (6). The remaining six patients with available imaging showed radiological abnormalities that either coincided with clinical relapse or remained stable without new MRI activity. Three patients had no available imaging during the RTX treatment period.

**Table 2:** Clinical timeline of the 10 RTX-resistant NMOSD patients.

Patient	Age at onset (years)	Age at first dose of RTX	RTX regimen	Duration of RTX therapy (months)	Age at switch (years)	Potential Precipitating Factors for Relapse	B cell level at relapse (%)	Memory B cell level at relapse (%)	Serum IL-6 (pg/mL) at relapse	Alternative therapies after RTX	Follow-up after switch (months)	Time to B cell re-appearance (≥ 1%) (months)	Adverse Events
1	23	28	100 mg+500 mg	12	29	None identified	0.2	0.01	Normal	Oral Prednisone 16mg/d	32	Not monitored	AIHA
2	51	55	100 mg × 3d	6	56	None identified	0.1	Unknown	Normal	MMF+LDS	47	15	None
3	12	13	500 mg	108	22	Infection (Legionella pneumophila IgM+)	0.2	0.03	Normal	MMF+LDS	40	20	None
4	22	41	100 mg × 3d	6	42	None identified	0.22	Unknown	Normal	MMF+LDS+IVIg	34	No re-appearance	TB
5	23	38	100 mg × 3d	48	42	None identified	0.0	0.04	Unknown	Ofatumumab	22	No re-appearance	None
6	24	49	100 mg × 3d	28	51	None identified	0.02	0.02	Normal	Inebilizumab	36	No re-appearance	None
7	13	22	100 mg × 3d	11	23	None identified	0.04	0.00	Normal	Inebilizumab	27	No re-appearance	None
8	24	31	100 mg × 3d	60	36	None identified	0.21	0.05	62.69	Satralizumab	23	No re-appearance	None
9	38	41	500 mg	36	44	None identified	0.0	0.03	15.93	Satralizumab	10	No re-appearance	None
10	22	41	100 mg × 3d	42	45	None identified	0.2	Unknown	Normal	Eculizumab	13	No re-appearance	None

RTX, rituximab; NMOSD, neuromyelitis optica spectrum disorder; EDSS, expanded disability status scale; B cell level at relapse, Proportion of B cells within peripheral blood mononuclear cells at relapse (%); Memory B cell level at relapse, Proportion of memory B cells within peripheral blood mononuclear cells at relapse (%); 100 mg+500 mg, dose of 100 mg on day 1 followed by 500 mg on day 2; 100 mg × 3d, 100 mg on three consecutive days; 500 mg, single 500 mg dose; LDS, Low-dose steroids (4 mg per day of oral prednisone); MMF, mycophenolate mofetil; IVIg, intravenous immunoglobulin; Re-appearance of B cells, B cells ≥ 1%; No re-appearance, B cell levels remained below 1% throughout the follow-up period; AIHA, autoimmune hemolytic anemia; TB, tuberculosis.

After RTX failure, patients transitioned to alternative therapies based on a combination of clinical, biomarker, and practical considerations. These included: (1) de-escalation strategies (n=4), primarily due to economic constraints, comprising oral prednisone alone (Patient 1, 16 mg/d), mycophenolate mofetil (MMF) with low-dose steroids (LDS) (Patient 2: MMF 1250 mg/d + prednisone 4 mg/d, Patient 3: MMF 1000 mg/d + prednisone 4 mg/d), and MMF + LDS + monthly IVIg (Patient 4: MMF 1000 mg/d + prednisone 4 mg/d); (2) anti-CD20 monoclonal antibody ofatumumab (n=1, Patient 5), chosen as newer monoclonal antibody agents were not yet commercially available at the time; (3) anti-CD19 monoclonal antibody inebilizumab (n=2, Patients 6 and 7), for patients without significant IL-6 elevation; (4) IL-6 receptor antagonist satralizumab (n=2, Patients 8 and 9), both of whom showed elevated serum IL-6 levels at relapse (62.69 and 15.93 pg/mL, respectively), whereas the other seven patients had normal levels (≤ 5.4 pg/mL), aligning the treatment choice with the underlying inflammatory signature; and (5) C5 complement inhibitor eculizumab (n=1, Patient 10), which was expensive and not covered by medical insurance at the time, limiting its accessibility to only a few patients. The time to B cell re-appearance after RTX cessation varied across patients: two patients on de-escalations (MMF + LDS) showed B cell recovery at 15 and 20 months post-switch, respectively, while the remaining eight patients—including those treated with ofatumumab, inebilizumab, satralizumab, or eculizumab—had no documented B cell re-appearance during follow-up. Notably, the patient who switched to ofatumumab (Patient 5) remained B cell depleted throughout ofatumumab therapy, including at the time of relapse, consistent with sustained CD20 suppression. However, given the retrospective nature of this study, B cell monitoring was not systematically protocolized, and the precise timing of recovery may not have been captured in all patients. The series was followed for a median of 28.4 months (range, 10-47) post-switch (**Table 2**).

**Figure 2:**
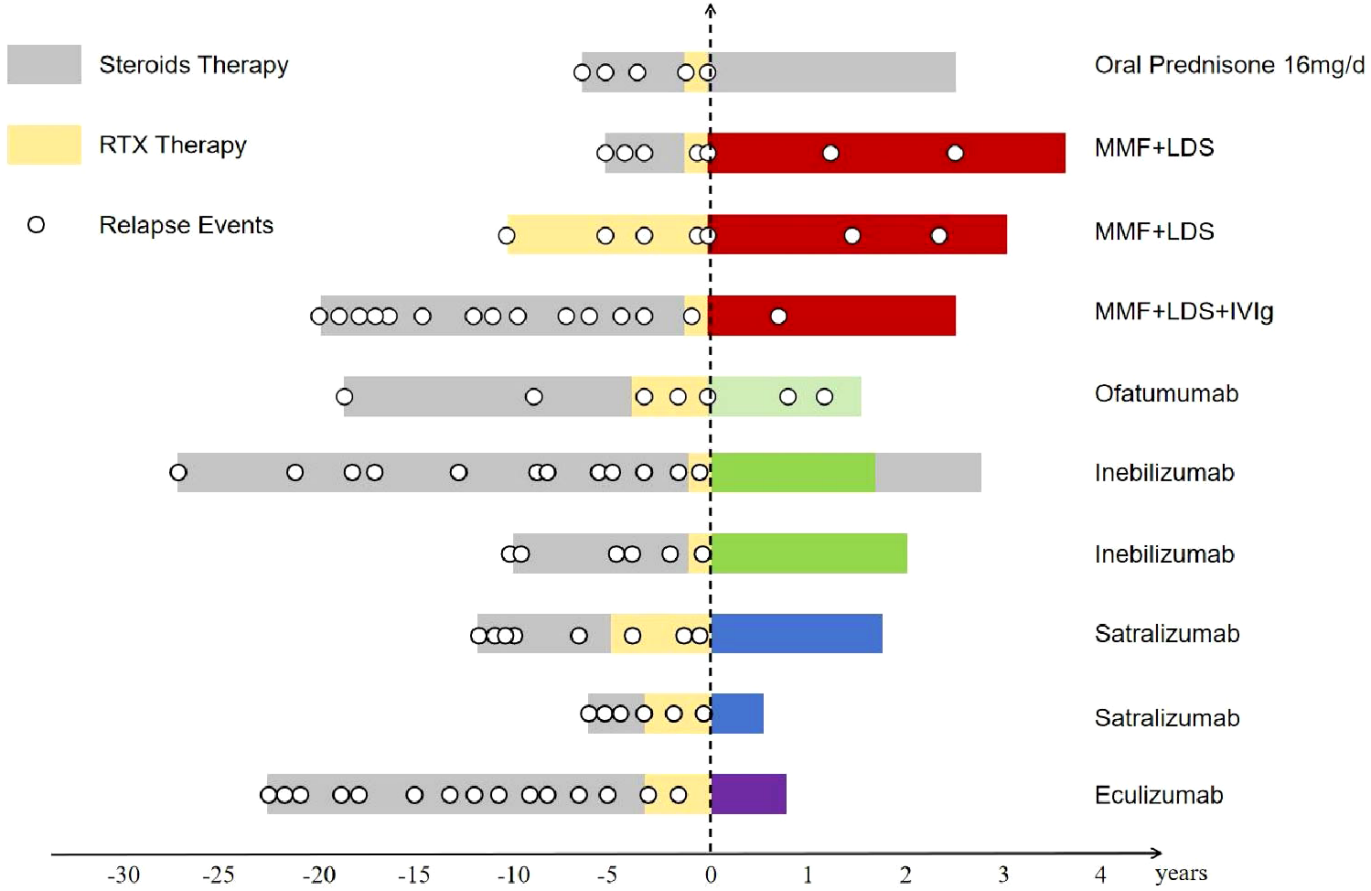
Treatment timeline and relapse events in 10 RTX-resistant NMOSD patients. Time zero (0) represents the point of switching from RTX to alternative therapies. Colored bars represent treatment duration; unfilled circles indicate relapses. Post-switch therapies include de-escalations (oral prednisone 16 mg/d, MMF + LDS, and MMF + LDS + IVIg), ofatumumab, inebilizumab, satralizumab, and eculizumab. RTX: rituximab; NMOSD, neuromyelitis optica spectrum disorder; LDS, Low-dose steroids (4 mg per day of oral prednisone); MMF, mycophenolate mofetil; IVIg, intravenous immunoglobulin.

All patients who transitioned to satralizumab, inebilizumab, or eculizumab achieved relapse-free survival (Patients 6-10). However, the patient treated with ofatumumab (Patient 5) relapsed. Three of four patients (75%) on de-escalations relapsed (Patient 2-4) at a median of 10 months post-switch (**Table 3**). Among patients who transitioned to targeted monoclonal antibody therapy, four showed improvement in EDSS scores, while two remained stable. In patients receiving de-escalation regimens, one improved, two remained stable, and one experienced EDSS worsening. A detailed description of each patient’s treatment timeline and relapse events is provided in **Figure 2**.

**Table 3:** EDSS scores and ARR before and after therapy switch in 10 RTX-resistant NMOSD patients.

Patient	Alternative therapy after RTX		Relapse after switch (Y/N)	Time to relapse (months)	EDSS before RTX	EDSS before switch	EDSS at last follow-up	ARR before switch	ARR after switch
1	de-escalations	Oral prednisone 16mg/d	No	–	4.0	4.0	4.0	1.00	0
2		MMF+LDS	Yes	15, 30	3.5	3.0	3.0	1.00	0.51
3		MMF+LDS	Yes	17, 28	3.0	3.5	3.0	0.50	0.60
4		MMF+LDS+IVIg	Yes	8	4.5	5.0	6.0	0.70	0.35
5	Anti-CD20	Ofatumumab	Yes	10, 13	4.5	7.0	4.5	0.26	1.09
6	Anti-CD19	Inebilizumab	No	–	4.5	3.5	3.0	0.44	0
7		Inebilizumab	No	–	3.0	4.5	3.0	0.58	0
8	IL-6R Antagonist	Satralizumab	No	–	3.5	4.0	3.0	0.67	0
9		Satralizumab	No	–	2.0	2.0	2.0	1.09	0
10	C5 Complement Inhibitor	Eculizumab	No	–	3.0	6.0	6.0	0.65	0

RTX, rituximab; NMOSD, neuromyelitis optica spectrum disorder; LDS, Low-dose steroids (4 mg per day of oral prednisone); MMF, mycophenolate mofetil; IVIg, intravenous immunoglobulin; Anti-CD20, anti-CD20 monoclonal antibody, targeting and depleting CD20+ B cells; Anti-CD19, Anti-CD19 monoclonal antibody, targeting and depleting CD19+ B cells.

Among the 10 patients who relapsed during RTX therapy, a potential precipitating factor prior to relapse was identified in only one patient (Patient 3), who tested positive for Legionella pneumophila IgM antibodies; no specific triggers were identified in the remaining nine patients. No significant adverse events were reported among patients who transitioned to ofatumumab, inebilizumab, satralizumab, or eculizumab. In contrast, those on de-escalations experienced notable safety signals, including one serious infection (tuberculosis in Patient 4) and one immune-mediated hematological event (autoimmune hemolytic anemia in Patient 1). The occurrence of serious events with de-escalations underscores the need for vigilant monitoring when using these broader agents.

## 4 Discussion

This retrospective case series describes 10 AQP4-IgG+ NMOSD patients who experienced relapse despite sustained peripheral B cell depletion (proportion of CD19^+^ B cells within peripheral blood mononuclear cells at relapse < 1% and CD27^+^ memory B cells < 0.05%) after more than six months of RTX therapy—a phenotype we define as “RTX resistance”. These 10 patients were identified from a total of 118 RTX-treated patients at our center, representing 8.5% of the treated population. This relatively low proportion suggests that RTX resistance is an uncommon but clinically challenging phenomenon that warrants further investigation. Due to the small sample size and descriptive nature of this study, the findings should be interpreted as hypothesis-generating rather than conclusive, and no comparative statistical analyses were performed. In this series, all five patients who switched to inebilizumab, satralizumab, or eculizumab remained relapse-free during follow-up. In contrast, relapse occurred in the patient switched to ofatumumab, an anti-CD20 monoclonal antibody that shares the same target (CD20) as RTX. Furthermore, three of the four patients on de-escalations experienced relapses. Our observations suggest that, in patients with RTX-resistant NMOSD, switching to monoclonal antibodies with novel mechanisms of action may offer better relapse prevention compared to de-escalation or sequential CD20-targeted therapy.

The relapse following ofatumumab, despite sustained B cell depletion, supports the concept of CD20-independent pathogenesis. RTX spares CD20-negative, AQP4-IgG-producing plasmablasts (1), whereas inebilizumab targets CD19, enabling depletion of these cells (12). Additionally, inebilizumab may offer an advantage (13) over RTX by not being limited by FCGR3A polymorphisms associated with relapse risk (11, 14, 15). In Patient 5, relapse occurred during ofatumumab therapy with B cells still < 1%, confirming that breakthrough disease can arise despite effective CD20 suppression. The failure of sequential CD20-targeted therapy further suggests that patients with RTX-resistant disease may not benefit from agents sharing the same mechanism of action. The two patients with elevated IL-6 levels at relapse (Patients 8 and 9) remained relapse-free after switching to satralizumab, an IL-6 receptor antagonist. By blocking IL-6 signaling—which dysregulates neutrophils and regulatory lymphocytes (16)—satralizumab disrupts a key pro-inflammatory axis in NMOSD pathogenesis (17), offering a strategic therapeutic alternative for patients failing B cell depletion therapy (18, 19). Eculizumab, a C5 complement inhibitor, acts downstream in the pathogenic pathway by inhibiting the terminal complement cascade, making it a valuable option even for patients who are RTX-resistant (20, 21).

The de-escalation strategies used in Patients 1-4 — oral prednisone alone (Patient 1, prednisone 16 mg/d), MMF + LDS (Patients 2 and 3, prednisone 4 mg/day), and MMF + LDS + monthly IVIg (Patient 4, prednisone 4 mg/day)—were primarily driven by practical and economic constraints, including drug availability, insurance coverage, and patient preference. Furthermore, long-term anti-CD20 therapy carries a risk of hypogammaglobulinemia (5), and de-escalation may be considered to mitigate this risk (22). Although the steroid doses in Patients 2-4 (prednisone 4 mg/day) align with guideline recommendations for long-term maintenance (≤ 7.5 mg/day) (14), Patient 1 received a higher dose (prednisone 16 mg/day) due to strong psychological dependence on corticosteroids. The MMF doses used in Patients 2-4 (1000–1250 mg/day) were also within the recommended range (1000–2000 mg/day) (14). However, de-escalation is not a preferred strategy in RTX-resistant NMOSD. A French registry study of 137 RTX de-escalations reported relapses across all regimens, with 9.2% of AQP4+ patients relapsing within 12 months; no group remained relapse-free except pregnancies (22). These findings underscore the inherent risk of de-escalation and align with our observation that three of the four patients on de-escalations experienced relapse. Thus, while de-escalation may be unavoidable in constrained settings, it should not be viewed as a therapeutic strategy of choice. In alignment with current consensus (8, 14, 23), approved monoclonal antibodies targeting novel mechanism— inebilizumab, satralizumab, and eculizumab—should be prioritized whenever accessible (24–27).

The management of RTX-resistant NMOSD is evolving beyond de-escalations, with our data supporting a strategic shift to approved non-CD20 targeted monoclonal antibodies. This approach is contextualized within a broader spectrum of emerging and alternative therapies reported in the literature. For chronic relapse prevention, targeted biological agents such as tocilizumab (anti-IL-6R) and the dual-target telitacicept (a TACI-Fc fusion protein) have demonstrated promise in small series by disrupting plasma cell survival and B cell activation (28). In contrast, for severe acute attacks, more intensive interventions like immunoadsorption (IA) and lymphocyte plasma exchange serve as rapid rescue therapies to remove pathogenic antibodies (29–31). Eculizumab also demonstrates outstanding potential as a salvage therapy. Among aggressive immunosuppressive options, cyclophosphamide (CTX) and double-filtration plasmapheresis (DFPP) are reserved for catastrophic cases (32). Looking forward, next-generation strategies such as anti-BCMA CAR T-cell therapy aim to achieve precise, long-term depletion of plasma cells, representing a potential paradigm shift towards sustained remission (33). While this expanding landscape offers multiple pathways, the approved monoclonal antibodies (eculizumab, inebilizumab, satralizumab) currently possess the most robust evidence base for sustained relapse prevention. Our findings support prioritizing targeted monoclonal antibodies over de-escalations for RTX-refractory NMOSD. Consequently, our findings help crystallize a treatment hierarchy that prioritizes mechanism-driven biologics for chronic management. Future research should focus on generating comparative effectiveness data, optimizing treatment sequencing, and developing biomarkers to guide personalized therapeutic selection in this complex clinical scenario.

This case series has several limitations. First, the small sample size (n=10) precludes definitive conclusions and limits the generalizability of the findings. Second, follow-up duration varied across patients, which may have influenced the detection of late-onset relapses or B cell recovery events. Third, although potential precipitating factors for post-switch relapse were assessed in all patients, subclinical triggers may have been underdetected due to the retrospective nature of data collection. Fourth, pharmacokinetic confounders, such as variability in RTX bioavailability or adherence, could not be fully accounted for. Despite these limitations, our findings reflect real-world clinical scenarios and provide hypothesis-generating data that may inform clinical decision-making. Larger, prospective studies with standardized protocols are warranted to validate these observations and guide optimal treatment strategies in RTX-resistant NMOSD.

## 5 Conclusion

In this descriptive case series, patients with RTX-resistant NMOSD who switched to inebilizumab, satralizumab, or eculizumab appeared to have favorable outcomes compared to those receiving de-escalation strategies or ofatumumab. De-escalation was employed only under constrained circumstances and is not endorsed as a preferred approach. These real-world observations provide hypothesis-generating data that may inform clinical decision-making, but require validation in larger, prospective cohorts.

## Data Availability

The original contributions presented in the study are included in the article/**Supplementary Material**. Further inquiries can be directed to the corresponding author.
