# Supplementary material for: Subsequent treatment strategies following rituximab-resistance in AQP4-IgG+ neuromyelitis optica spectrum disorder: a case series
Source: Front Immunol. 2026 Apr 16;17:1762535. doi: 10.3389/fimmu.2026.1762535 (PMC13128675; doi:10.3389/fimmu.2026.1762535)
Supplement: Supplementary file 1 [file Table1.docx]

**Table S1. MRI findings in RTX-resistant NMOSD patients.**

| **Patient** | **MRI lesions newly detected (compared with previous imaging)** | |
| --- | --- | --- |
|  | **Within 3 Months Before Clinical Relapse** | **At Clinical Relapse** |
| 1 | Not performed | Bilateral optic nerves and optic chiasm showed slight T2 hyperintensity with patchy mild enhancement |
| 2 | Not performed | Enlarged T4-5 spinal cord lesion |
| 3 | Unchanged | Left posterior optic nerve thickening and enhancement |
| 4 | *No available* | *No available* |
| 5 | *No available* | *No available* |
| 6 | Not performed | Unchanged |
| 7 | *No available* | *No available* |
| 8 | Not performed | T3-T10 abnormal signal with heterogeneous enhancement |
| 9 | **New abnormal enhancement at C2-3** | No available |
| 10 | Not performed | Unchanged |

Abbreviations: MRI: magnetic resonance imaging; RTX: rituximab; NMOSD: neuromyelitis optica spectrum disorder. Not performed: No MRI was obtained within 3 months prior to clinical relapse. Unchanged: No new or enlarging lesions compared with the most recent previous MRI. *No available*: MRI was performed at an outside hospital and the imaging data could not be obtained for review.


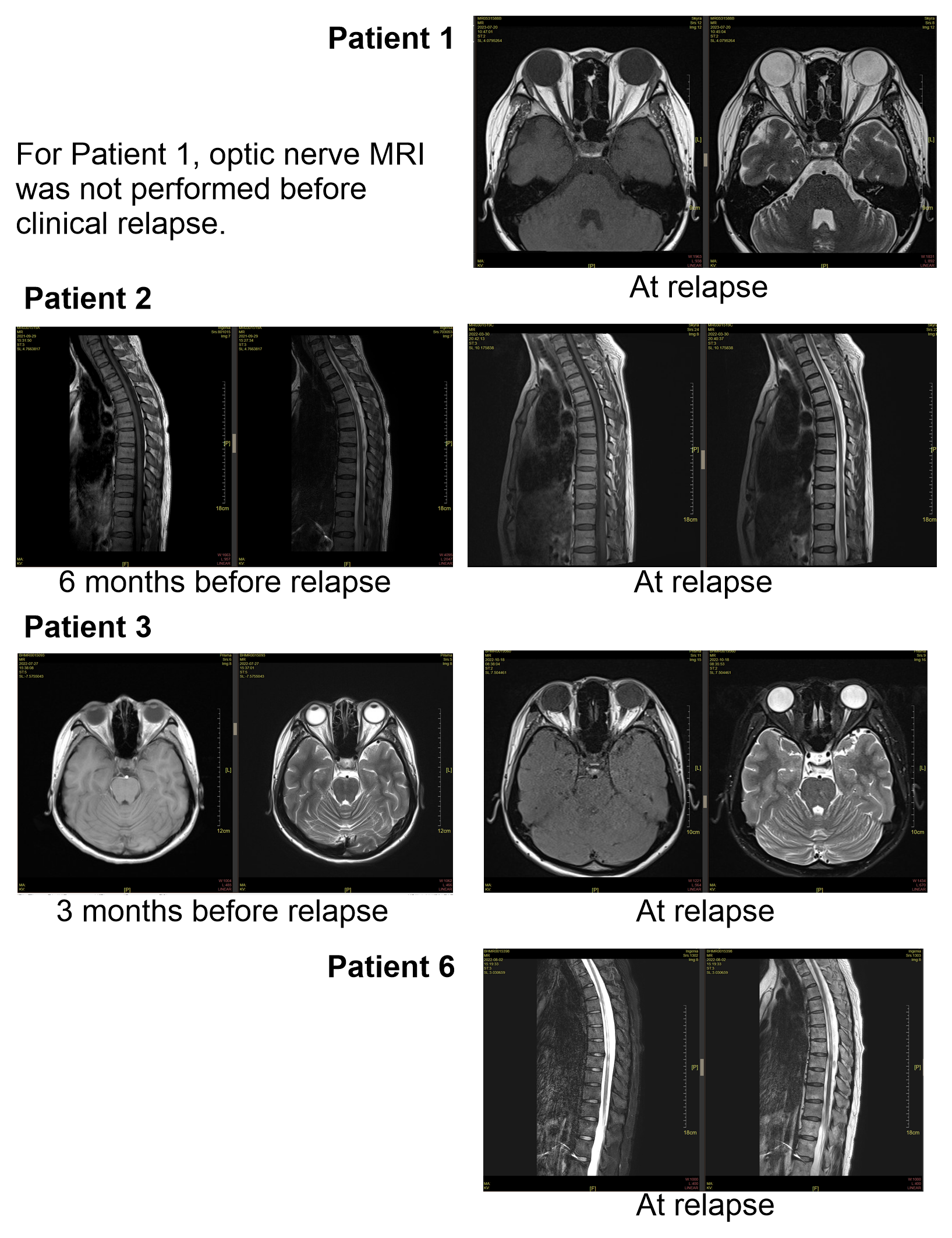

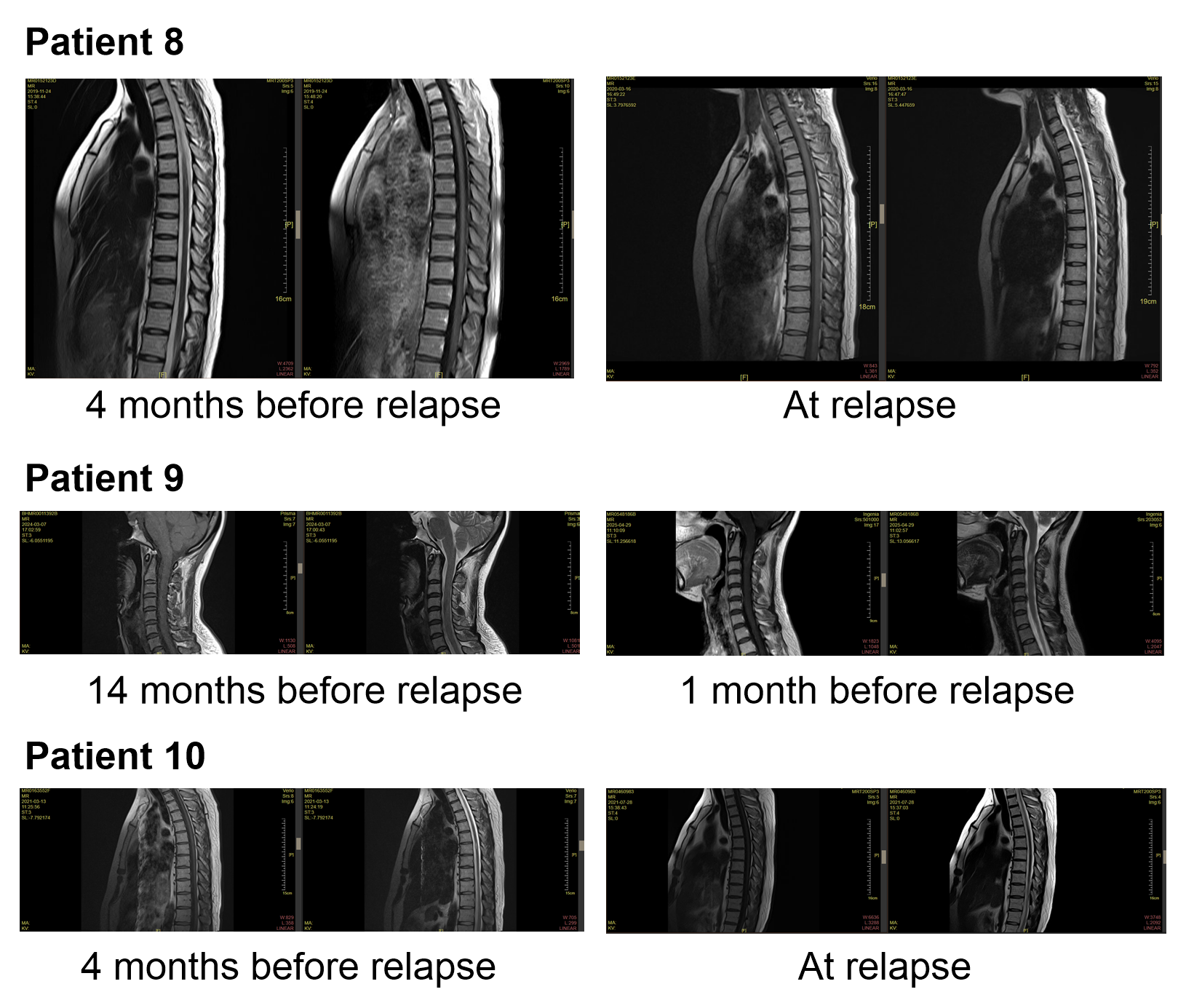


**Figure S1. MRI findings in RTX-resistant NMOSD patients.**

For each patient, MRI images are presented at two time points: the most recent MRI prior to relapse and the MRI at clinical relapse. For each time point, T1-weighted images are shown on the left and T2-weighted images on the right.
